# Bidirectional Microglia–Neuron Communication in Health and Disease

**DOI:** 10.3389/fncel.2018.00323

**Published:** 2018-09-27

**Authors:** Zsuzsanna Szepesi, Oscar Manouchehrian, Sara Bachiller, Tomas Deierborg

**Affiliations:** Experimental Neuroinflammation Laboratory, Department of Experimental Medical Science, Lund University, Lund, Sweden

**Keywords:** fractalkine, extracellular vesicles, neuroinflammation, neurodegeneration, microglia, neuron, cytokines

## Abstract

Microglia are ramified cells that exhibit highly motile processes, which continuously survey the brain parenchyma and react to any insult to the CNS homeostasis. Although microglia have long been recognized as a crucial player in generating and maintaining inflammatory responses in the CNS, now it has become clear, that their function are much more diverse, particularly in the healthy brain. The innate immune response and phagocytosis represent only a little segment of microglia functional repertoire that also includes maintenance of biochemical homeostasis, neuronal circuit maturation during development and experience-dependent remodeling of neuronal circuits in the adult brain. Being equipped by numerous receptors and cell surface molecules microglia can perform bidirectional interactions with other cell types in the CNS. There is accumulating evidence showing that neurons inform microglia about their status and thus are capable of controlling microglial activation and motility while microglia also modulate neuronal activities. This review addresses the topic: how microglia communicate with other cell types in the brain, including fractalkine signaling, secreted soluble factors and extracellular vesicles. We summarize the current state of knowledge of physiological role and function of microglia during brain development and in the mature brain and further highlight microglial contribution to brain pathologies such as Alzheimer’s and Parkinson’s disease, brain ischemia, traumatic brain injury, brain tumor as well as neuropsychiatric diseases (depression, bipolar disorder, and schizophrenia).

## Microglia – Guards of the CNS Homeostasis

Microglia are the resident immune cells of the central nervous system, which represent about 5–12% of total CNS cells in the healthy brain and the spinal cord. Microglia are derived from the myeloid precursors cells in the embryonic yolk sac and they travel to the area of the developing CNS during early embryogenesis (Schulz et al., 2012; Ginhoux et al., 2013; Kierdorf and Prinz, 2013; Ginhoux and Prinz, 2015; Wieghofer and Prinz, 2016; Hoeffel and Ginhoux, 2018). Microglial progenitors are already present around the neural tube at embryonic day 9 in mice, and from the fifth gestational week in humans. After neural entry, microglia progenitors migrate through the developing nerve tissue and undergo massive proliferation before they reach their final density (Ginhoux et al., 2010; Verney et al., 2010). Embryonic microglia develop into highly ramified mature microglia via regulatory factors such as Pu.1, IL-34, and CSF-1 (Schulz et al., 2012; Kierdorf et al., 2013). Microglia are broadly distributed throughout the CNS although quantity and location among species varies (Lawson et al., 1990; Mittelbronn et al., 2001). Despite their broad distribution, microglia represent a rather heterogeneous community with different subpopulation based on specific brain regions. Each microglia subpopulation develop unique features and they can be distinguished by capacities and functions (Gertig and Hanisch, 2014; Neiva et al., 2014; Doorn et al., 2015; Grabert et al., 2016; De Biase et al., 2017). Microglia are considered to be the resident macrophages in the CNS and are long-lived, self-renewing cells, autonomous from peripheral monocytes that normally do not enter the brain.

Phenotypically, ramified microglia have been described as ‘quiescent’ or remaining in a ‘resting’ state. However, these views radically changed after *in vivo* imaging studies have revealed the extraordinary active nature of microglia processes in the healthy brain (Davalos et al., 2005; Nimmerjahn et al., 2005). Microglia are constantly restless and their processes undergo continuous cycles of extension and withdrawal and *de novo* formation to scan their environment for disruptions in brain homeostasis thereby ‘resting’ microglia are able to acquire numerous phenotypes. Each microglia process seems to have a defined territory and they are able to scan their environment within several hours. Microglia process movement is systematically aimed at synapses to monitor and regulate neuronal activity, indicating the presence of specific signaling mechanisms that direct microglial processes to synapses (Li et al., 2012, 2013; Dissing-Olesen et al., 2014). When microglia detect ‘danger’ signals that compromise CNS homeostasis- for instance through pathogen recognition receptors- they rapidly change their appearance by shortening of cellular processes, enlargement of their soma and they transform into a reactive phenotype. Reactive microglia can further evolve into phagocytic or amoeboid microglia that completely lack cellular processes (Streit, 2000; Stence et al., 2001; Ransohoff and Perry, 2009; Graeber and Streit, 2010; Kettenmann et al., 2011). In response to CNS injury or infections, microglia are able to upregulate expression of many cell surface receptors including toll-like receptors (TLRs), phagocytic receptors (CR3, CR4), scavenger receptors (CD36, CD91) and release various complement factors (Tian et al., 2012). However, microglia activation does not refer to a single phenotype, and a continuum of microglia activation is rather considered. They are able to acquiring numerous phenotypes upon activation ranging from a phagocytic to an antigen presenting phenotype that mainly depends on the type of stimuli provided in their environment (Town et al., 2005; Schwartz et al., 2006; Boche et al., 2013). Once activated, microglia can be potent immune effector cells and initiate both innate and adaptive immune responses and produce a number of cytokines, chemokines and growth factors (Ransohoff and Perry, 2009; Loane and Kumar, 2016; von Bernhardi et al., 2016). Microglia accept a wide variety of inputs and they are also able to provide an appropriate response to a multitude of reactions. Therefore, microglia activation is considered to be a flexible and adaptive process rather than being stereotypic and granted (Schwartz et al., 2006; Hanisch and Kettenmann, 2007; Deczkowska et al., 2018). Importantly, microglia are also capable of morphological remodeling without any indication of an insult or neurodegeneration. Chronic stress, enhanced glutamatergic neurotransmission or light deprivation can lead to hyper-ramification of microglia and more frequent neuron–microglia contacts (Tremblay et al., 2010; Hinwood et al., 2012; Walker et al., 2013; Torres-Platas et al., 2014; Yirmiya et al., 2015). These observations suggest that there is information transmission between microglia and neurons and microglia are continuously informed about the actual activity or state of neurons in their vicinity.

## Reciprocal Neuron–Microglia Communication

Microglia are in close contact with neurons as well as oligodendrocytes and astrocytes. Astrocytes are the most abundant glial cells in the CNS and they show significant contribution to synapse formation, maintenance and elimination, thus regulating the overall architecture and activity of neuronal circuits. Astrocytes perform direct contacts with their neuronal pre- and postsynaptic counterparts and they also release soluble factors to modulate synaptic transmission of both excitatory and inhibitory synapses. This led to the concept of the ‘tripartite synapse,’ a synapse composed of two neurons and an astrocyte as an integrated functional unit (Perea et al., 2009; Nistico et al., 2017; Farhy-Tselnicker and Allen, 2018). The tripartite synapses are supplemented by microglia, which are specially attracted by synapse activity, location of crosstalk between neurons and glial cells. In the healthy brain, microglia exhibit an actively repressed ‘surveying’ phenotype that is dependent on a dynamic crosstalk between microglia and neurons (Biber et al., 2007; Kettenmann et al., 2011; Kierdorf and Prinz, 2017). It has been proposed that the removal of this neuronal derived inhibitory control represents a type of danger signal for microglia, indicating that neuronal function is impaired and leads to alterations of microglia morphology and function. The reciprocal neuron–microglia communication is mediated by numerous soluble factors, extracellular vesicles (EVs) as well as contact-dependent mechanisms, and is essential for adaptive neuroplasticity and learning (Posfai et al., 2018).

## Classical Cell-To-Cell Communication

**FIGURE 1:**
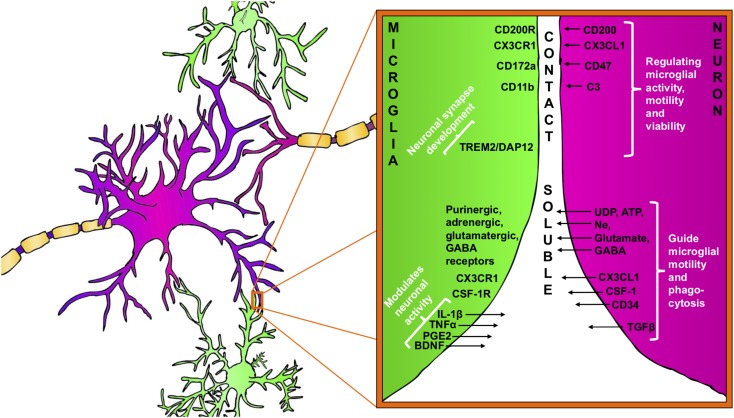
Bidirectional signaling between microglia (green) and neuron (purple). Neuron–microglia communication is mediated by receptor–ligand interactions as well as by various soluble factors. Microglia are equipped with a group of surface receptors, which trigger signals and regulate specific microglia function like phagocytosis, motility and viability. Many of the receptor ligands, such as CX3CR1, CD200R, and CD172a are released or expressed on the surface of neurons. Receptor–ligand interactions represent a classical contact dependent communication between microglia and neurons. Microglia and neurons reciprocally release soluble factors that can modulate cell functions and promote tissue homeostasis.

**FIGURE 2:**
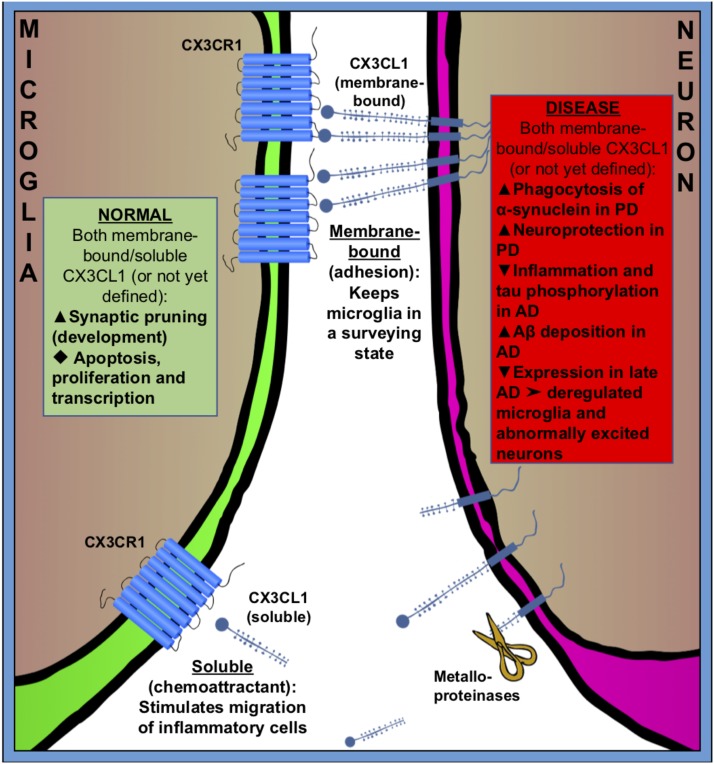
Fractalkine (CX3CR1–CX3CL1) signaling between microglia (green) and neuron (purple) in normal (green square) and pathologic (red square) states. CX3CL1 is either membrane-bound or cleaved by metalloproteinases to become soluble. Membrane bound CX3CL1 is an OFF-signal, that keeps microglia in a surveying state, and soluble CX3CL1 is believed to act as a chemoattractant, stimulating migration of inflammatory cells. Fractalkine signaling has many roles in both healthy and pathologic conditions. Black triangles indicate increase (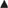), decrease (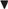) or not specified (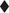).

In the healthy brain microglia remain remarkably quiescent or ‘unactivated’ while undertaking surveillance roles. It is now recognized that this ‘unactivated’ state is under the control, at least in part, of neuronal factors, including CD200 and fractalkine (CX3CL1) as the most studied cell-to-cell proteins (Eyo and Wu, 2013) (**Figures 1**, **2**). CD200 is a glycoprotein widely found on the cell membranes of neurons, astrocytes and oligodendrocytes in the CNS (Wright et al., 2001; Barclay et al., 2002; Koning et al., 2009). The target receptor of CD200, CD200R, is only found on macrophages and microglia (Hernangomez et al., 2012). When neuronal CD200 interacts with microglial CD200R, the microglia will be kept in its inactivated, resting state (Hoek et al., 2000; Biber et al., 2007) thus CD200 signaling plays a critical role in neuronal protection. Defaults in CD200 signaling have been observed in several neuroinflammatory conditions like in multiple sclerosis (MS), Alzheimer’s disease, or in the aging brain (Hernangomez et al., 2012; Varnum et al., 2015; Xie et al., 2017).

The lack of CD200 in an experimental animal model for MS (EAE) revealed an accelerated progression of the MS symptoms, indicating that microglia attain a dysregulated activation in absence of CD200-signaling (Hoek et al., 2000).

Fractalkine, also known as CX3CL1, is a unique chemokine that is constitutively expressed in neurons (mainly in the prosencephalon) (Tarozzo et al., 2003). The fractalkine receptor, CX3CR1, is predominantly found on microglia and on neurons in less amount in the normal CNS (Harrison et al., 1998; Meucci et al., 2000; Hatori et al., 2002; Paolicelli et al., 2014) and CX3CL1–CX3CR1 signaling has been considered a neuronal ‘off signal’ that keeps microglia in a surveying phenotype (Nishiyori et al., 1998; Maciejewski-Lenoir et al., 1999; Biber et al., 2007). It should be noted that under chronic inflammatory conditions, glial cells also express CX3CL1 (Hughes et al., 2002).

CX3CL1 is found both as membrane-bound, to neuronal membranes, and cleaved (soluble) into the extracellular space (**Figure 2**). The cleavage of CX3CL1 in its heavily glycosylated chemokine domain is performed by metalloproteinases (MMPs) (Garton et al., 2001). CX3CL1 bound to the membrane is believed to be important for adhesion, while the soluble CX3CL1 acts a chemo-attractant, recruiting inflammatory cells (Bazan et al., 1997; Schall, 1997; Mizoue et al., 2001). The G-protein-linked receptor CX3CR1 triggers several intracellular secondary messengers, such as P13K, AKT, and NF-κB, and thus regulates apoptotic, proliferative, transcriptional and migratory functions in microglia (Al-Aoukaty et al., 1998; Chandrasekar et al., 2003). The fractalkine receptor is expressed by other cell types than microglia, such as monocytes, dendritic cells and natural killer cells, but because these cells don’t frequently cross the brain–blood barrier (BBB) in healthy brains, microglia are believed to be the main recipient of CX3CL1-signaling in the normal CNS (Imai et al., 1997; Harrison et al., 1998; Truman et al., 2008; Mizutani et al., 2012; Wolf et al., 2013).

Expression of fractalkine increases in the CNS during embryonic and postnatal maturation and promotes microglial recruitment to neuronal circuits that need rewiring during a period of activity-dependent remodeling. In return, microglia modulate neuronal function and survival via the release of trophic factors (Ueno et al., 2013). In the absence of fractalkine signaling, microglial colonization is impaired in the somatosensory, motor cortex and the hippocampus during the early postnatal development (Paolicelli and Gross, 2011; Hoshiko et al., 2012) (**Figure 2**).

Replacing the CX3XR1-gene with GFP, in the CX3CR1-GFP knock-in mouse line (Jung et al., 2000), has been proved to be a very useful tool to study microglial function and fractalkine signaling in the healthy brain. Experimental paradigms comparing CX3CR1-GFP homozygous mice (CX3CR1^-/-^) that entirely lack CX3CR1, and therefore of fractalkine signaling in the brain, with heterozygotes (CX3CR1^-/+^) proved to be extremely useful strategy to disclose the molecular players in neuron–microglia communication in a non-invasive way related to fractalkine signaling.

Besides fractalkine, numerous other chemokines are produced and released by neurons and glial cells (by both astrocytes and microglia) in the CNS. CCL2/MCP-1 is expressed in the neurons principally during pathological conditions as most studies have shown, although some observations support the notion of a constitutive neuronal CCL2 expression (Coughlan et al., 2000; Banisadr et al., 2005; Conductier et al., 2010). The receptor for CCL2 is found in microglia and its activation trigger chemotaxis of cultured microglia. An induction of neuronal CCL2 expression was described in response to various types of injury and degeneration such as ischemia, Alzheimer’s disease, MS, axonal injury, amyotrophic lateral sclerosis (ALS) or peripheral nerve injury (Barna et al., 1994; Che et al., 2001; Pang et al., 2001; Rancan et al., 2001; Schreiber et al., 2001; Baron et al., 2005; Bose and Cho, 2013; Perner et al., 2018). The chemokine CCL2 is also produced by glial cells (Barna et al., 1994; Hanisch, 2002; He et al., 2016) as perivascular astrocytes have been identified as the most common and predominant source of CCL2 in the CNS in various neuroinflammatory conditions (Andjelkovic et al., 2002; Guillemin et al., 2003). Another chemokine, CXCL12/SDF-1 (stromal cell-derived factor 1) and its receptor CXCR4 have been reported to be produced and expressed predominately by astrocytes and neurons. Astrocytic activation of CXCR4 can lead to the release of pro-inflammatory cytokines and prostaglandins, and it is able to trigger glutamate exocytosis from astrocytes thereby modulate neuronal activity. A unique chemokine, CCL21, is exclusively expressed on endangered neurons after injury and activate microglia through chemokine receptor CXCR3 (Biber et al., 2001; Dijkstra et al., 2004; van Weering et al., 2010).

Overall, chemokines are able to control neuron–microglia interactions through diverse intracellular signaling upon chemokine receptor activation and contribute to a variety of cellular functions such as modulation of neurotransmission, regulating cell survival and BBB permeability, exert neuroprotection, migration of neuronal progenitors, stem cells and axonal sprouting (Mackay, 2001; Mellado et al., 2001; Reaux-Le Goazigo et al., 2013; Zheng et al., 2017).

Furthermore, many other inhibitory receptors/molecules can be found on microglial surfaces that interact with ligands from neurons (both secreted and membrane-bound). An example is the integrin CD47, which communicates a “don’t eat me”-signal to microglial CD172a (van Beek et al., 2005; Biber et al., 2007) (**Figure 1**) by recruiting tyrosine-protein phosphatases SHP-1 and SHP-2, down-regulating phagocytosis as well as by increasing the synthesis of TGF-β (reviewed in Griffiths et al., 2007).

## Fractalkine Signaling Regulates Synapse Development and Synaptic Plasticity

During the first week of postnatal life vast amounts of synapses are generated during an intensive period of synaptogenesis and this is also when microglia density reaches its maximum (Dalmau et al., 2003). Neuronal networks are refined by synaptic pruning, a selective and activity-driven process that eliminates redundant, developing synapses. Microglia make contacts with presynaptic axon terminals and postsynaptic dendritic spines that can be followed by a complement-dependent phagocytosis and elimination (Wakselman et al., 2008; Tremblay and Majewska, 2011; Cunningham et al., 2013; Jung and Chung, 2018).

Fractalkine signaling has been implicated in microglial pruning of dendritic spines during normal brain development (**Figure 2**). Paolicelli and Gross (2011) and Paolicelli et al. (2011) showed that a decreased microglial density in the hippocampus of CX3CR^-^/^-^ mice was associated with a temporary boost in dendritic spine numbers on pyramidal neurons and an excess of immature synapses compared with CX3CR^-^/^+^ littermates. It is suggested that microglia lacking the CX3CR1 receptor fail to recognize synapses displaying fractalkine. Comparing homozygous CX3CL1^-/-^ and heterozygous CX3CL1^-/+^ mice, microglial fractalkine signaling was shown to play a crucial role in the maturation of excitatory glutamatergic synapses and remodeling of neuronal circuits during postnatal development (Bertollini et al., 2006; Ragozzino et al., 2006; Hoshiko et al., 2012; Zhan et al., 2014) (**Figure 2**); although, the role of neuron–microglial crosstalk in this context is still not fully understood. In the developing barrel field of the somatosensory cortex, fractalkine (CX3CL1/CX3CR1) signaling has been also shown to regulate microglial recruitment to the location where developing thalamocortical synapses are concentrated (i.e., barrel centers). Hoshiko et al. (2012) showed that microglia entry into the barrel centers is temporally delayed in CX3CR1^-/-^ mice compared with WTs at the developmental stage, when fractalkine is overexpressed within the barrels. CX3CR1 deficiency also delays the functional maturation of glutamate receptors and results in a significantly lower proportion of postsynaptic AMPARs in CX3CR1^-/-^ than in CX3CR1^-/+^ mice. Moreover, the developmental switch from GluN2B to GluN2A-containing NMDA receptors, which are known to occur during the early weeks of postnatal development of thalamocortical area, was delayed in CX3CR1^-/-^ mice but this delay was only transient. These observations support to the notion that microglia influence synaptic maturation during development and fractalkine signaling deficiency induced a transient impairment in the maturation of glutamate receptor functional expression at thalamocortical synapses. However, the exact mechanism by which microglial cells influence synapse maturation is complex and it is not fully understood. Microglia release several signaling molecules known to modulate the functional expression of glutamate receptors. For instance, glia-derived TNF-α facilitates AMPA receptors trafficking and membrane insertion (Beattie et al., 2002; Stellwagen et al., 2005). Also, TNF-α specifically controls the glutamate release step of gliotransmission in the hippocampal dentate gyrus (Santello et al., 2011). Similarly, brain-derived neurotrophic factor (BDNF) released by microglia has been demonstrated to modulate spine density and the expression of AMPA and NMDA receptors in cortical neurons of adult mice (Parkhurst et al., 2013). Pascual et al. (2012) have showed *in vitro* that microglia are able to regulate synaptic neurotransmission by releasing ATP, which binds to P2Y1R located on astrocytes and enhances excitatory postsynaptic current. Also, microglia regulate synaptic functions and neuronal development of cultured neurons through the interactions of the microglia-released interleukin 10 (IL-10) with IL-10 receptors (IL-10R) expressed by neurons (Lim et al., 2013). Thereby, fractalkine-dependent recruitment of microglia within the barrel centers might induce the release of microglia-derived signaling factors necessary for alterations of the functional expression of glutamate receptors at thalamocortical synapses.

Moreover, a lack of CX3CL1-signaling, and therefore a weakened crosstalk between neurons and microglia, affects transmission efficiency in synapses of the adult brain. It was observed that CX3CR1^-/-^ had fewer multi-synaptic boutons in the hippocampus CA1 region compared to wild-type littermates indicating a lasting impairment of synaptic connectivity (Zhan et al., 2014). Changes in long-term potentiation (LTP) have been found in CX3CR1^-/-^ mice, and were accompanied with deficits in behavior related to learning and memory (Rogers et al., 2011). The Rotarod test revealed that motor control in particular was negatively affected in these mice, compared to control animals. However, the CX3CR1^-/-^ mice also appear to have deficits in associative learning, measured by fear-conditioning tests, and in hippocampal-dependent memory formation – as revealed with Morris water maze. These findings indicate that fractalkine signaling plays a significant role in learning and memory, and the effects appear to be dependent on the activity of interleukin 1β (IL-1β). The expression of IL-1β was increased in both heterozygous and homozygous CX3CR1 knockouts, and the hippocampal infusion of the IL-1β antagonist, IL-1ra, reversed the behavioral deficits in the CX3CR1^-/-^ mice, indicating that IL-1β, released from microglia, could mediate these effects (Rogers et al., 2011). The dose-dependent inhibitory effects of IL-1β on LTP in the hippocampus are supported by other studies and this inhibition is consistent with the high distribution of IL-1R1 on hippocampal neurons (Lynch, 2015). The inhibitory effects of IL-1β in hippocampus have been linked with stimulation of the stress-activated kinases, p38 and JNK (O’Donnell et al., 2000; Vereker et al., 2000). Interestingly, the anti-inflammatory IL-10, which could be also released by microglia, has been demonstrated to antagonize certain actions of IL-1β. IL-10 is able to abrogate the IL-1β -induced inhibition of glutamate release and LTP and its stimulatory effect on JNK signaling (Kelly et al., 2001).

Although fractalkine signaling has a significant role in synapse pruning, it is not the only signaling pathway involved in this complex process. Microglial complement 3 receptor (C3R) has been also determined participating in developmental synaptic pruning. In the healthy developing brain, C1q, the protein that initiates the classical complement pathway of the complement system, promotes activation of C3, which opsonizes subsets of synapses for elimination and promote microglial engulfment and phagocytosis of synaptic elements. This process is significantly downregulated in the mature brain (Stevens et al., 2007; Schafer et al., 2012; Bialas and Stevens, 2013). However, in an early phase of AD this normal developmental synaptic pruning pathway can be locally reactivated and it mediates synapse loss. Hong et al. (2016) demonstrated that in an AD-like mouse strain, C1q was increased and co-localized with synapses even before visible plaque deposition, and blocking C1q, C3 or the microglial receptor CR3, decreased microglial phagocytosis and early synapse loss. The possible relationships between fractalkine signaling and the classical complement pathway could further complicate the topic of developmental and pathological synaptic pruning and it demands more research. Microglia-synapse interactions and synapse removal has also been discovered in many brain pathologies. Synaptic dysfunction and loss, as well as microglial activation, are early events in neurodegenerative diseases such as AD and Huntington’s disease (Perry and O’Connor, 2010). Despite the extensive research of the topic, the question is remaining: are microglia initiating synapse defects and loss in neurodegenerative diseases or is their activation just a secondary phenomenon during pathogenesis?

Apart from the fractalkine and complement signaling, a role for microglial DAP12 (DNAX-activation protein 12) has also been reported in the development of functional neuronal synapses. Intriguingly, in genetically deficient DAP12 mice, developmental apoptosis of neurons was decreased (Wakselman et al., 2008) but synaptic plasticity was enhanced (Roumier et al., 2004). Interestingly, DAP12 function has also been linked to TREM2 (triggering receptor expressed in myeloid cells 2) both are expressed on microglia in the brain and form a receptor-adaptor complex. TREM2/DAP12 signaling is known to regulate microglial phagocytosis and connected to numerous other intracellular signaling pathways implicated in regulation of synaptic plasticity (Kiialainen et al., 2005; Hsieh et al., 2009) (**Figure 1**).

Overall, the proper microglia functions and their interactions with synaptic elements are considered to be instrumental for appropriate neuronal development and also support the homeostasis of neuronal networks in adults.

## Soluble Factors Mediating Neuron–Microglia Communication

Neurons are able to release further immune-related soluble factors that bind to cognate receptors on microglia and promote specific microglia phenotype (**Figure 1**). These comprise neurotrophins, neuropeptides, neurotransmitters, anti-inflammatory cytokines and chemokines (Biber et al., 2007; Kerschensteiner et al., 2009).

For instance, the multifunctional cytokine, transforming growth factor beta (TGF-β), which is expressed by both neurons and glial cells, has been recognized as a vital regulator for microglia differentiation that promotes a unique transcription profile and surface structure of adult microglia (Butovsky et al., 2014). Also, TGF-β is a potent regulator of cytotoxicity and neuroinflammation in the nervous system (John et al., 2003; Saud et al., 2005). Its importance is to down-regulate microglial responses as showed by increased microglial activity and neuronal loss in the brains of TGF-β-deficient mice (Brionne et al., 2003). Smad pathway has been considered as one of the main signal transduction pathway activated by TGF-β receptors, which is responsible for the regulatory and neuroprotective effects of TGF-β. TGF-β-Smad signaling is involved in the induction of the quiescent phenotype of microglia within the CNS (Abutbul et al., 2012). Binding of TGF-β and its receptor induces ligand dependent assembly of a heteromeric receptor complex, receptor-kinase activation and subsequent phosphorylation and activation of SMAD proteins. SMADs are transcriptional regulators that accumulate in the nucleus and directly regulate gene transcription to evoke cell-type-specific and context-dependent transcriptional programs (Schmierer and Hill, 2007). Besides Smad proteins, there are additional signaling pathways activated by TGF-β, including ERK, p38 and PI3K those can mediate synergistic or antagonistic effects thereby, the responses of activated microglia are the end result of interactions of different signaling pathways (Derynck and Zhang, 2003; Lee et al., 2007).

Furthermore, CD45, a leukocyte common antigen, is constitutively expressed at moderate levels on microglia. Its activation leads to inhibition of microglia activity via negative regulation of the Src/p44/42 MAPK cascade (Tan et al., 2000). The endogenous ligand of CD45 is CD22 has been found at neuronal membranes and neurons secrete CD22 following neuronal injury in order to inhibit microglial proinflammatory cytokine production (Mott et al., 2004).

Electrically active neurons can suppress the interferon-gamma (IFNγ)-induced increase in the expression of pro-inflammatory MHC class II molecules on microglia in the intact CNS (Vass and Lassmann, 1990) and in cultured hippocampal slices (Neumann et al., 1996). The neurotransmitter glutamate, which is released during synaptic activity, is involved in the regulation of microglial cells via glutamate receptors, including metabotropic receptors (Fazio et al., 2018). Glutamate does not necessarily act directly on microglial cells. Instead neuronal released trophic factors such as neurotrophin-3 (NT-3), BDNF and nerve growth factor (NGF) were identified as activity-dependent regulators of microglial MHC class II expression and induction of pro-inflammatory molecules. Neuron-derived glutamate stimulates BDNF and NGF production or release while the regulation of NT3 production by synaptic activity appears to be indirect. Neurotrophins bind to two different classes of receptors: the tyrosine kinase receptors (trkA, trkB, or trkC) or the p75 neurotrophin receptor (also known as the low-affinity NGF receptor). Neumann et al. (1998) and Neumann (2001) have showed that NGF and, to a lower extent, NT3 but not BDNF acted directly on isolated microglia through the p75 neurotrophin receptor as MHC class II inducibility could be enhanced by neutralizing locally released neurotrophins (NGF, BDNF, and NT3) or blocking the p75 neurotrophin receptor. Thus, neurotrophins secreted by electrically active neurons are able to control the antigen-presenting potential of microglia, which is mediated partly via the p75 neurotrophin receptor.

Several neurotransmitters have modulatory effects on microglial activity and proliferation that are related to local neurochemical environment and could differ across various brain regions (McCluskey and Lampson, 2001). As mentioned, glutamate can exert an inhibitory regulation of microglial cells via metabotropic glutamate receptors (mGluRs; Fazio et al., 2018). Microglia also express GABA-B receptors which activation strongly decreases the LPS-induced secretion of certain but not all inflammatory cytokines (Kuhn et al., 2004). Glycine, which is the other inhibitory neurotransmitter, also attenuates the production of inflammatory cytokines and the phagocytic activity of brain macrophages (Zeilhofer, 2008). Similarly, noradrenalin reduces the LPS-stimulated release of NO, IL-6, and TNF-α. Dopamine has also been reported to modulate the activation, proliferation, and cytokine release in immune cells (Sarkar et al., 2010). Both D1 and D2 dopamine receptors mediate anti-inflammatory effects and inhibit neuroinflammation and attenuate brain injury after intracerebral hemorrhage in mice (Zhang et al., 2015; Wang et al., 2018).

In response to any disturbance of their microenvironment, microglia are able to respond rapidly and these responses are mediated, in part, by neuron-released nucleotides such as ATP and UDP (**Figure 1**). The role of the nucleotides for microglial activation and proliferation has been established, particularly during the early phase of brain injury (Haynes et al., 2006; Ohsawa et al., 2012; Ulmann et al., 2013). The chemotactic properties of ATP are mediated by microglial P2Y12 purinergic receptors and the absence of P2Y12 leads to impaired microglia process motility during injury. Davalos et al. (2005) confirmed the relevance of ATP-induced microglial chemotaxis *in vivo* in the mouse cortex. The authors showed that laser-induced injury to brain tissue resulted in robust microglial branch extension toward the site of injury and the process of the chemotaxis was abolished by an ATP/ADP degrading enzyme (Davalos et al., 2005). ATP-induced microglial chemotaxis was then confirmed in acute mouse brain slices in the mouse spinal cord (Chen et al., 2010; Dibaj et al., 2010) and retina (Fontainhas et al., 2011) as well as in other animal models. Another purinergic receptor, P2Y6, is also expressed by microglia and UDP signaling through P2Y6 receptors triggered microglial phagocytosis following hippocampal excitotoxicity (Koizumi et al., 2007). Extensive and prolonged neuronal release of glutamate directly leads to neuronal death but also serves as activation signal for microglia through a variety of microglial glutamate receptors like AMPA, kainate, and mGluRs (Biber et al., 1999; Hagino et al., 2004). It has been demonstrated that the activation of microglia mGluR2 triggers TNF-α-induced neurotoxicity through activation of TNF receptor-1 and facilitating the activation of caspase-3. Microglia also released FasL, which further potentiated TNF-α neurotoxicity after mGlu2 stimulation (Taylor et al., 2005).

In return, microglia influence and modulate neuronal function by the release of soluble factors, including cytokines, prostaglandins and neurotrophic factors, which bind to neuronal receptors. IL-1β, prostaglandins (PGE_2_), BDNF and tumor necrosis factor-alpha (TNF-α) are often released by microglia in response to variations in neuron-derived signals.

Brain-derived neurotrophic factor plays and important role in neuronal survival and differentiation and as a neuromodulator, directly involved in the control of neuronal activity and synaptic plasticity (Santos et al., 2010) (**Figure 1**). The neuromodulatory effect of BDNF has been recognized both on glutamatergic and GABAergic synapses in the CNS (Gottmann et al., 2009). Apart from neuron and astroglia, microglia also release BDNF as it was first shown in microglia cultures (Elkabes et al., 1996) and then confirmed in different regions of the CNS during the course of various neurological disorders such as traumatic injury, Parkinson’s disease, MS, and neuropathic pain (Batchelor et al., 1999; Knott et al., 2002; Stadelmann et al., 2002; Trang et al., 2011; Song et al., 2016). The synthesis and release of BDNF in microglia appear to be tightly associated with the activation of ATP sensitive purinergic receptors, such as P2X4R. The activation of P2X4R leads a significant intracellular Ca^2+^ flow and the downstream activation of signaling pathways like p38 MAP kinase, which controls the synthesis and release of BDNF (Trang et al., 2009). Then, microglia-derived BDNF rapidly downregulates K^+^-Cl^-^ co-transporter KCC2 expression in neuronal membranes through tyrosine kinase B receptor (TrkB) which, disrupting Cl^-^ homeostasis and the strength of GABA_A_^-^ and glycine receptor-mediated inhibition thereby leads to an altered neuronal network activity (Ferrini and De Koninck, 2013). Although the low levels of microglia-derived cytokines are demonstrated to support homeostatic neuroplasticity, these signaling pathways could be further augmented during inflammatory conditions and mediate neurotoxicity (Stellwagen et al., 2005; Parkhurst et al., 2013; Pribiag and Stellwagen, 2014; Stellwagen and Lewitus, 2014).

## Microglial Communication by Extracellular Vesicles

Extracellular vesicles are recently discovered way of communication between cells in the CNS that are providing new insights into the brain physiology and pathophysiology of several diseases (Basso and Bonetto, 2016; Rufino-Ramos et al., 2017; Paolicelli et al., 2018; Trotta et al., 2018). Indeed, we found recently that inflammatory stimuli to microglia leads to distinct populations of released EVs, both in terms of size and protein content (Yang et al., 2018a).

In one of the earliest study on EVs in the CNS, membrane exovesicles were identified as vehicles for spreading morphogens through epithelia during *Drosophila melanogaster* development (Greco et al., 2001). Since then the influence of EVs in tissue development has also been observed in the developing mouse brain and undifferentiated neuronal culture (Marzesco et al., 2005; Faure et al., 2006; Marzesco, 2013).

The family of EVs contains different types of vesicles that are distinguishable by their size, biological origin and function. The EV family comprises exosomes (40–120 nm) that are released from multivesicular endosomes, microvesicles (100–1,000 nm) that are budding from the plasma membrane and apoptotic bodies (800–5,000 nm) that are released by cells during apoptosis (Cocucci et al., 2009; Gyorgy et al., 2011; Colombo et al., 2014; Cocucci and Meldolesi, 2015; Dozio and Sanchez, 2017).

All brain cells, including neurons (Faure et al., 2006; Lachenal et al., 2011) astrocytes (Dickens et al., 2017) microglia (Hooper et al., 2012; Prada et al., 2013; Glebov et al., 2015) and oligodendrocytes (Kramer-Albers et al., 2007; Fitzner et al., 2011; Fruhbeis et al., 2013) secrete EVs. EVs contain different bioactive compounds including cell surface receptors, mitochondrial and cytosolic proteins, metabolic enzymes and genetic materials such as microRNAs and mRNAs (Abels and Breakefield, 2016; Dozio and Sanchez, 2017; Prada et al., 2018). Additionally, EVs could carry pathological markers, such as α-synuclein, tau, amyloid beta (Aβ) (Rajendran et al., 2006; Ngolab et al., 2017; Valdinocci et al., 2017) and pathogenic prion proteins (Schneider and Simons, 2013; Vilette et al., 2018) as well as huntingtin (Zhang et al., 2016; Deng et al., 2017) that implicate exchange of EVs in pathological conditions. EVs are able to influence the behavior of recipient cells in multiple ways: they may transfer receptors and/or bioactive lipids between cells; they can modulate functional target cells by delivering intracellular proteins or transferring mRNA; and may act as signaling complexes through the stimulation of target cells (Basso and Bonetto, 2016). In the CNS, there is an extensive cross talk between neurons and microglia and alongside the actual cell-to-cell contact and the cellular release of soluble factors, microglia and neurons can communicate by bidirectional release of EVs, which permits an exchange of a wide range of biomolecules across long distances (Garzetti et al., 2014; Lai et al., 2014; Rajendran et al., 2014; Zaborowski et al., 2015; Budnik et al., 2016; Kramer-Albers and Hill, 2016).

Microvesicles released by microglia are known to differ in shape and size (100 nm – 1 mm) and they are able to modulate the activity of neighboring microglial population and/or neurons in the surroundings. For instance, microglia-derived EVs can increase glutamate release at the presynaptic sites of neuronal synapses thereby enhances excitatory synaptic transmission. The stimulation of synaptic activity occurs via enhanced neuronal sphingolipid metabolism (Antonucci et al., 2012). Gabrielli et al. (2015) demonstrated that microglial EVs are also able to modulate synaptic transmission through the modulation of the endocannabinoid system. Endocannabinoids are secreted through microglial EVs that have on their surface *N*-arachidonoylethanolamine (AEA), which can stimulate type-1 cannabinoid receptors (CB_1_), and inhibit presynaptic transmission of GABAergic neurons.

Microvesicles shedding from microglia is an intriguing topic but little is yet known and therefore warrants further investigations. Extracellular ATP is a key stimulant for vesicles shedding from microglia via the P2X7 ATP receptor (P2X7R) that activates the p38 MAPK cascade through src kinase-mediated phosphorylation. Phosphorylated p38 triggers microvesicle shedding and IL-1β release from glia cells via a process that requires activation of acid sphingomyelinases (Bianco et al., 2005, 2009). Additionally, IL-1β-loaded microvesicles released by microglia via P2X7-p38 pathway can enhance the sensitivity of mechanical allodynia and thermal hyperalgesia induced by nerve injury (Li et al., 2017). Microglial P2X7R is also responsible for the release of glyceraldehyde-3-phosphate dehydrogenase (GAPDH) into the extracellular space, which could be involved in the regulation of neuroinflammation and/or neuritogenesis in the brain (Takenouchi et al., 2015). Others and we have applied a proteomic approach to characterize the proteome of EVs released by microglia. Several proteins involved in protein translation, transcription, cell adhesion/extracellular matrix organization, autophagy-lysosomal pathway and cellular metabolism, that may influence the response of target cells to EVs were identified (Drago et al., 2017; Yang et al., 2018a). Interestingly, upon LPS stimulation proteins related to RNA processing and protein translation were upregulated in the EVs (Yang et al., 2018a). Furthermore, glial microvesicles can contain purine nucleoside phosphorylase, a crucial enzyme in purine metabolism which converts ribonucleosides into purine bases and it can be released into the extracellular space through P2X_7_R activation, indicating that glial cells may support neuronal activity by maintaining the homeostasis of the purinergic system (Pena-Altamira et al., 2018). Serotonin can also stimulate exosome release both in primary microglia cultures and BV2 cell lines (Glebov et al., 2015). Additionally, stimulation with lipopolysaccharide induces microvesicles release from microglia that can carry proinflammatory mediators (Jablonski et al., 2016; Kumar et al., 2017). We found LPS stimulation of microglia specifically upregulated TNF, and to a lower extent IL-6, in EVs released (Yang et al., 2018a).

Accumulated research confirms the role of microglia-released EVs in neurodegenerative conditions. For instance, shedding of microglia-derived microvesicles was demonstrated after traumatic brain injury (TBI), ischemic stroke, spinal cord injury and in neuropathic pain (Kumar et al., 2017; Huang et al., 2018; Mondello et al., 2018; Osier et al., 2018). Microvesicle from the animals with TBI are loaded with pro-inflammatory mediators (IL-1β and microRNA-155) and can activate additional glial cells that may contribute to progressive neuroinflammatory response in the injured brain (Kumar et al., 2017). ALS is a fatal disease characterized by progressive degeneration of motor neurons and by the formation of inclusions consisting of SOD1 and TDP-43 in motor neurons. Recently, exosomes from astrocytes have been noted that are able to transfer misfolded SOD1 to spinal neurons and subsequently cause selective motor neuron death (Basso et al., 2013). In AD, toxic Aβ and hyperphosphorylated tau can be spread between cells by exosomes and therefore they have been known for contributing to apoptosis and neuronal loss. Rajendran et al. (2006), have shown (in N2a and Hela cells) that Aβ is present in multivesicular bodies (MVBs), and it is released into the extracellular space in exosomes upon MVBs fusion with the plasma membrane. Extracellular Aβ also activates microglia, and activated microglia-shed microvesicles into their environment. α-Synuclein exposure to murine microglial cell line BV2 increased the secretion of EVs enriched in TNF-α and MHCII molecules and promotes neuronal apoptosis (Chang et al., 2013). Furthermore, Joshi et al. (2014) have demonstrated that microglia-shed microvesicles promote the extracellular formation of highly toxic soluble form of Aβ thereby induces neurotoxicity. They have also found that microglia generate neurotoxic species following Aβ internalization, which are delivered to neurons possibly on the external membrane of microvesicles, which leads to neuronal damage.

On the other hand, microglia are able to clear Aβ by phagocytosis of the EVs loaded with Aβ. Yuyama et al. (2015) and Yuyama and Igarashi (2016, 2017) have demonstrated that neuronal exosomes can sequester Aβ through its abundant glycosphingolipids and then Aβ could be taken up and digested by microglia. The administration of neuronal exosomes into the brain of APP transgenic mice results in a decreased amyloid deposition and neuronal exosomes can effectively ameliorates AD pathology (Yuyama et al., 2015; Yuyama and Igarashi, 2016, 2017). Tamboli et al. (2010) have described another possible way by which microglia-shed exosomes could digest extracellular Aβ and thereby promote amyloid clearance. The insulin degrading enzyme (IDE) that is known to be effectively degrade extracellular amyloid deposits was found to be associated and released by exosomes shed by microglia (Tamboli et al., 2010). Microglial exosomes are also involved in the spread of tau pathology as was shown by numerous studies (Saman et al., 2012; Medina and Avila, 2014; Asai et al., 2015; Wang et al., 2017; DeLeo and Ikezu, 2018; Guix et al., 2018). Thus, in the context of neurodegeneration, microglia-derived EVs could play complex and also controversial roles. Microglial EVs have been demonstrated to spread toxic and mutant proteins, while other studies indicated their positive impact on protein aggregates clearance and regulation of neuronal viability. In line with this, various studies demonstrated that microglia activation in early stages of AD pathogenesis could be neuroprotective. However, in the late stage of the disease, microglial MVs have been found responsible for the transportation and distribution of soluble toxic proteins like Aβ and α-synuclein peptides, and to promote the spread of the disease (Benilova et al., 2012; Tofaris, 2017; Croese and Furlan, 2018; Zhang S. et al., 2018).

## Microglia in Pathological Conditions

It has become evident that microglial cells are involved in essentially all brain diseases ranging from AD and PD, TBI, brain ischemia and psychiatric diseases such as schizophrenia (Wohleb, 2016; Joe et al., 2018; Perea et al., 2018; Stephenson et al., 2018). The outcome of many pathologies of the CNS appear to rely heavily on the activity of microglia, including their release of cytokines, chemokines and growth factors (Ransohoff and Cardona, 2010). Importantly, microglial functions are largely dependent on the type of activation stimuli, time after stimulation and local factors during pathological conditions. Microglia responses are not inevitably neurotoxic and various neuroprotective effects of activated microglia have been observed *in vivo* (Turrin and Rivest, 2006; Szalay et al., 2016). In general, rapid and acute activation of microglia is associated with inflammatory changes but these are designed to combat the immediate insult and ultimately return the tissue homeostasis. This acute reaction could therefore be considered to be neuroprotective in the longer perspective, unless the acute response is augmented and prolonged. Persistent microglial activation with the associated increase in expression of inflammatory cytokines and chemokines, accompanied by recruitment of peripheral cells into the brain, is typically characterized as detrimental chronic neuroinflammation (Lynch, 2009; Lynch et al., 2010). This prolonged and chronic microglia activation is considered to be neurotoxic and can impair neuronal activity. This ambivalent role of microglia in neurodegenerative diseases has been extensively reviewed in the literature, searching for a consensus amidst conflicting data and summating whether the activity of microglia is helpful or not in pathologies of the brain (Hu et al., 2012; Jiang et al., 2014; Loane and Kumar, 2016; Szalay et al., 2016; Du et al., 2017).

Neuroinflammation occurs not only in the brain, but also in the retina and optic nerve – which are outgrowths from the diencephalon and thus considered parts of the CNS (London et al., 2013). In fact, retinal homeostasis is dependent on many of the same bidirectional signaling pathways between brain microglia and neurons, such as fractalkine signaling (Karlstetter et al., 2015). The retina and the brain are affected in several neurodegenerative diseases and because they are similar, they respond similarly to any disturbances of tissue homeostasis and share common pathogenic mechanisms (London et al., 2013). For instance the extracellular deposits of beta-amyloid and intraneuronal accumulation of hyperphosphorylated tau protein reported in AD are also found in the retina and optic nerve. Similarly, PD involves the degeneration of dopaminergic cells in the retina (Ramirez et al., 2017). Excellent reviews are available covering the topic of microglia involvement in retinal physiology and pathology (Wang J.W. et al., 2016; Akassoglou et al., 2017; Jin et al., 2017; Rathnasamy et al., 2018).

## Contribution Factors of Microglia and Fractalkine Signaling in AD and PD

Alzheimer’s disease is a progressive neurodegenerative disorder characterized by the plaque-forming accumulation of amyloid-β (Aβ) and the deposition of neurofibrillary tangles (NFTs, composed of hyper-phosphorylated tau protein) within the brain parenchyma. Beta-amyloidosis is a result from an imbalance production versus clearance of Aβ peptides. Microglia are able to engulf and phagocyte the extracellular Aβ via stimulation of triggering-receptor-expressed on myeloid cells 2 (TREM2). TREM2 is a cell surface protein that is selectively and highly expressed by microglia and it is linked to an anti-inflammatory phenotype (Colonna and Facchetti, 2003; Mecca et al., 2018). TREM2 interacts with the adaptor protein DNAX-activating protein of 12-kDa (DAP12), which initiate intracellular signal transduction pathways that regulate microglial phagocytosis and activation. TREM2 is critical for microglial phagocytosis of for example, cellular debris in order to maintain tissue homeostasis (Neumann and Takahashi, 2007). In AD condition, TREM2-deficient microglia demonstrated a reduced uptake of Aβ-lipoprotein complexes *in vitro* (Yeh et al., 2016) and a less effective Aβ internalization *in vivo* (Wang et al., 2015; Wang Y. et al., 2016; Yuan et al., 2016). Microglia also have an ability to build a protective barrier around amyloid deposits, that is composed of tightly packed and thereby less toxic amyloid fibrils (Condello et al., 2015).

On the other hand, microglia have significant role in complement-mediated synapse loss in AD (Hong et al., 2016; Fonseca et al., 2017; Jiang and Bhaskar, 2017). In line with this, disabled microglial proliferation and microglial depletion protects against synapse loss in amyloidosis mouse models but does not necessarily have effect on the formation and maintenance of β-amyloid plaques (Grathwohl et al., 2009; Olmos-Alonso et al., 2016; Spangenberg et al., 2016). Complement activation also appears to exacerbate tau pathology and induces NFTs in AD mouse models via unclear mechanisms (Britschgi and Wyss-Coray, 2007; Fonseca et al., 2009). Asai et al. (2015) have showed that microglia are able to spread tau pathology across the brain by microglial uptake and exosomal release of tau in a mouse model of tauopathy. In addition to synapse elimination and aggravation of tau pathology, activated microglia release proinflammatory mediators in response to extracellular protein aggregates and thereby causing harm to neurons (Heneka et al., 2013; Shi et al., 2017).

Microglial fractalkine signaling plays variable roles in different stages of AD pathogenesis in association with neuroinflammation, neurotoxicity, and synaptic plasticity (Chen et al., 2016; Zhang L. et al., 2018). The neuronal soluble CX3CL1 is likely to keep the microglial phenotype in a rather neuroprotective state by acting on CX3CR1 in microglia, since the disruption of CX3CL1–CX3CR1 signaling leads to dysregulate microglial responses and neuronal damage (Cardona et al., 2006; Febinger et al., 2015) (**Figure 2**). Indeed, hAPP-CX3CR1^-/-^ mice as well as hTau-CX3CR1^-/-^ mice showed increased expression of inflammatory factors, enhanced tau phosphorylation, and exacerbated neuronal dysfunction and cognitive deficits (Bhaskar et al., 2010; Cho et al., 2011) (**Figure 2**). However, others have demonstrated that both APP-PS1/CX3CR1^-/-^ and CRND8/CX3CR1^-/-^ mice showed reduction in Aβ deposition with increased number of microglia (Lee et al., 2010; Liu et al., 2010). Moreover, in some cases the suppression of CX3CL1–CX3CR1 alleviated Aβ-induced neurotoxicity and memory deficiency (Wu et al., 2013; Dworzak et al., 2015). Additionally, the level of plasma soluble CX3CL1 was found to be significantly greater in the patients with mild to moderate AD than in the patients with severe AD, and the level of CX3CL1 is inversely correlated to AD severity (Kim et al., 2008), suggesting an early neuroinflammatory role in AD pathogenesis. Thus, CX3CL1/CX3CR1 signaling may play a beneficial role in controlling AD progression by inhibiting the inflammation and tau phosphorylation but at a cost of the increased Aβ deposition. One possible hypothesis is that, in early AD the intra-neuronal Aβ accumulation causes a mild decrease in neuron–microglia crosstalk via CX3CL1–CX3CR1 signaling that leads to an enhanced microglial phagocytosis of Aβ while resulting in tau hyper-phosphorylation. Later as AD is progressing, the communication between neurons and microglia is further exacerbated and the CX3CL1–CX3CR1 signaling is severely downgraded that gives rise to deregulated microglia and abnormally excited neuron, which leads to neuron damage and loss. Indeed, we have found remarkable early activation of inflammatory pathways in microglia at 6 weeks of age, before plaque deposition, in the 5xFAD mouse model of AD using a proteomic approach (Boza-Serrano et al., 2018).

Parkinson’s disease is characterized by the selective loss of dopaminergic neurons in substantia nigra. The pathological hallmarks are the formation of Lewy-bodies and intraneuronal protein inclusions mainly consist of α-synuclein. Microglial activation and neuroinflammation clearly contribute to neurodegeneration seen in PD, although the exact cellular mechanisms are not known yet. Neuron–microglia communication via fractalkine signaling provides an effective endogenous mechanism to moderate microglia activation and suppress the release of pro-inflammatory factors during the course of the disease (Grimmig et al., 2016). Maintaining or enhancing CX3CL1-CX3CR1 communication has been proved to be neuroprotective in multiple rodent models of PD (Pabon et al., 2011; Morganti et al., 2012; Nash et al., 2015). However, the action of fractalkine is intricate and regulated on multiple levels; for example, the CX3CR1^-/-^ mice have been demonstrated an enhanced Aβ phagocytosis and at the same time a decline in α-synuclein degradation (Thome et al., 2015) (**Figure 2**). It remains to be determined if this action is mediated by membrane bound fractalkine or the cleaved, soluble form.

## Microglia in Traumatic Brain Injury

Injuries to the CNS, including TBI, are the leading causes of death and severe disability for people under 40 years of age in the developed world (Donat et al., 2017). The clinical consequences of TBIs are very diverse including immediate death, complete recovery and permanent cognitive, emotional and physical impairments. TBIs are major risk factors for dementia (Donat et al., 2017; Saber et al., 2017) and chronic traumatic encephalitis (CTE) (Omalu, 2014). The primary injury of TBIs, caused by trauma to the head which for example might stretch, compress or tear blood vessels and axons, is not the only reason why patients die or are disabled (Prins et al., 2013). A lot of the damage is also caused by the secondary events such as edema, metabolic and blood flow disturbances, free radical formation, glutamate excitotoxicity, blood–brain barrier breakage, as well as neuroinflammation, i.e., activation of microglia and subsequent recruitment of peripheral leukocytes (Prins et al., 2013; Donat et al., 2017; Saber et al., 2017). Microglial activation in this context is multifactorial, e.g., primed by pro-inflammatory cytokines (Donat et al., 2017) or triggered through purinergic receptors by ATP released from necrotic neurons (Davalos et al., 2005; Fang et al., 2009), and could persist for years if not resolved (Braun et al., 2017). The methods for investigating the role of microglial activity following TBI, have been utilizing the same mouse models as have been discussed earlier in this review. Subjecting CX3CR1^GFP/GFP^ mice to experimental TBI have enabled studies of the fractalkine axis between microglia and neurons in a context of TBI (Febinger et al., 2015; Erturk et al., 2016; Zanier et al., 2016). Similar methods have also been applied for exploring the role of other signaling pathways in TBI, such as TREM2/DAP12, etc. (Saber et al., 2017). However, while these models could be beneficial in understanding the role of specific molecular pathways, it remains difficult to specify their cellular source, which could be microglial or peripheral macrophages (Sewell et al., 2004).

However, microglial signaling in neurodegeneration caused by TBI remains controversial (Bennett and Brody, 2014). A study by Bennett and Brody (2014) showed that in a mouse model of repetitive closed skull TBI (rcTBI) the partial depletion of Cd11b^+^ microglia did not affect axon degeneration at 7 or 14 days post injury (Bennett and Brody, 2014). Another study observed a big therapeutic effect of deleting or inhibiting complement system proteins C3 and C5 after traumatic brain cryoinjury – which was not attributed directly to the activity of microglia – but instead to a lesser invasion of neutrophils (Sewell et al., 2004).

So far, four peer reviewed studies have investigated the effects of the absence or partial absence of fractalkine signaling after experimental TBI, with some conflicting results (Febinger et al., 2015; Erturk et al., 2016; Zanier et al., 2016; Makinde et al., 2017). In one study, CX3CR1^GFP/GFP^ mice demonstrated a decrease of pro-inflammatory cytokines after the controlled cortical impact (CCI) model of TBI (Febinger et al., 2015). However, in another investigation with CX3CR1^-/-^ mice subjected to CCI, the trends in cytokine expression were not as clear (Zanier et al., 2016), although in both studies, early after insult, the mice lacking CX3CR1 performed the neuroscore test better than WT controls (Febinger et al., 2015; Zanier et al., 2016). This effect was reversed at a later stage (Febinger et al., 2015; Zanier et al., 2016), indicating that fractalkine signaling might exert negative influence at an early stage, but protective at later stages after TBI. It should be noted that, while fractalkine signaling is important in microglia–neuron bidirectional communication, CX3CR1 is also expressed in peripheral macrophages, and could contribute to their infiltration into the CNS (Erturk et al., 2016). A third study examined the effects of CCI in mice missing one allele of CX3CR1 and found that the female mice, but not the male mice, showed significantly better symptoms post-injury, including less neurodegeneration, leukocyte infiltration and cognitive deficits (Erturk et al., 2016). Here it was also shown that WT mice revealed neurodegeneration one year after insult, even in areas far from the lesion, indicating that targeting chronic fractalkine signaling is a promising, albeit sex-specific, way of combating detrimental events after TBI (Erturk et al., 2016). A fourth study subjected CX3CR1 null mice to CCI and observed that neutrophil infiltration was significantly reduced (Makinde et al., 2017).

The receptor adaptor complex TREM2/DAP12, important in synapse formation (Filipello et al., 2018), is also involved in microglial activation and phagocytosis (Mecca et al., 2018). A massive upregulation of TREM2 has been demonstrated in a mouse model of TBI and the absence of TREM2 was shown to improve hippocampal survival and cognition, lower disinhibitory behavior, as well as to decrease the immune cell activation throughout the brain, except in proximity to the lesion (Saber et al., 2017). However, the TREM2-dependent immune cell activity, believed to be negative in the TBI setting, was not primarily associated with microglia, but rather with peripherally derived macrophages (Saber et al., 2017).

TBI is associated with a wide range of deleterious events for cerebral vasculature and tissues that cause immune reactions and leukocyte infiltration in the brain (Saber et al., 2017; Monson et al., 2018). The activity of macrophages appears to be a potential therapeutic target in different TBI settings in adult, but not young, rodents (Febinger et al., 2015; Erturk et al., 2016; Hanlon et al., 2016; Zanier et al., 2016; Chhor et al., 2017; Saber et al., 2017). However, it appears that the peripherally derived macrophages, rather than macrophages derived from the resident microglial population, are the primary targets for attenuating detrimental effects after TBI (Bennett and Brody, 2014; Morganti et al., 2015; Saber et al., 2017). More research is required to elucidate the specific role of microglia and microglial pathways in the very multi-facetted context of TBI.

## Microglia–Neuron Communication in Brain Ischemia

Brain ischemia, or stroke, is one of the leading cause to death and disability in adults (American Heart Association, 2017). Brain ischemia is typically characterized as focal or global brain ischemia. The most common type of brain ischemia is stroke (focal ischemia), which to 85% is related to an occlusion of a cerebral artery or to less extent (15%) rupture of an cerebral artery and few treatment options is currently available and then related to clot removal or to resolve to clot in the immediate phase (Siegel et al., 2017). In global ischemia, the entire brain is ischemic, where most common condition leading to global brain ischemia is cardiac arrest with successful resuscitation (Joundi et al., 2016). In this section, we will focus on focal ischemia, which is characterized by an ischemic core, which in the acute phase is surrounded by a penumbra region, with impaired function, and injured tissue that is amenable for tissue protection. Neuroinflammation and microglia reactions are involved in all stages of the ischemic cascade: from the acute event leading to the first neuronal cell death to later stages of parenchymal processing including phagocytosis of cell debris and tissue remodeling. In this section, we will focus on the direct interaction between microglia–neuron, i.e., CD200 and fractalkine. Other recent reviews cover the broader aspect of inflammation in stroke and ischemia pathogenesis (Dirnagl, 2012; Benakis et al., 2014; Chamorro et al., 2016). As mentioned, several messengers are involved in the communication between microglia and neurons, for example cytokines and purines. The glycoprotein CD200 is mainly expressed by neurons and its receptor CD200R is expressed on myeloid cells including microglia. This interaction is involved in maintaining microglial cells in a quiescent homeostatic stage. Lower expression of CD200 has been related to proinflammatory activation of microglia and increased influx of inflammatory cells into the brain (Denieffe et al., 2013). Autocrine release of CD200 from microglia is also suggested to keep the microglia in an alternative/non-inflammatory activation state, involving IL-4 signaling (Yi et al., 2012). The endocannabinoid anandamide (AEA) which can be released by microglia in neuroinflammatory conditions and have an anti-inflammatory effect in stroke (Capettini et al., 2012), is also reported as a mechanism for upregulation of CD200R on microglia (Hernangomez et al., 2012). Additionally, peroxisome proliferator-activated receptor gamma (PPAR-γ), which is a transcription factor controlling the inflammatory response, has been reported to regulate CD200 and CD200R1 gene expression, which could be related to the neuroprotective action of PPAR-γ agonists (Dentesano et al., 2014). Recently, Yang et al. (2018b) investigated the acute effect, up to 48 h after ischemia, of CD200 following permanent focal ischemia in mice. They found CD200 expression on neurons, but not on microglia, and a negative correlation with neuronal death in cortical tissue. Injection of recombinant CD200 intracerebroventricular, performed right after pMCAO induction, reduced microglial activation and expression of cytokine TNF, IL-1β, and IL-10 (Yang et al., 2018b). To be able to use the beneficial effect of CD200 signaling in ischemic stroke, Kong et al. (2018) used human mesenchymal stem cells with high CD200 expression from human placenta which they transplanted intracerebrally 24 h after transient middle cerebral artery occlusion (tMCAO). Interestingly, they report reduced microglia activation in the infarct boundary area, smaller infarct and improved behavior. Silencing of CD200 in the mesenchymal stem cells also reduced the inflammatory response in BV2 microglia when applying a co-culture system (Kong et al., 2018). Using a similar rat stroke model, Matsumoto et al. (2015) investigated if the CD200 could be used to distinguish M1 or M2 macrophage/microglia in the infarct at 7 days after ischemia, but were not able to make this distinction in M1/M2 based on CD200 expression. However, using rat macrophages Hayakawa et al. (2016b) used CD200 stimulation induced alternative activation (M2) via CREB-C/EBP-beta signaling.

Focal white matter ischemia, where a small ischemic lesion can generate large neurological deficits, has also been studied in the context of CD200 signaling using endothelin injections in mice (Hayakawa et al., 2016a). Hayakawa et al. (2016a) used CD200-Fc treatment to target CD200R and found a reduction in the macrophage phagocytosis of oligodendrocyte progenitors, which was related to reduced TLR4 expression in macrophages. Indeed, they could even detect an enhancement of remyelination following CD200-Fc treatment (Hayakawa et al., 2016a). Microglia studies of CD200-deficient mice reveal increased classical activation, which could be related to increased infiltration of T cells and macrophages (Denieffe et al., 2013). Using CD200-deficient mice, or overexpression of CD200, using conditional systems in various experimental stroke models with long-term recovery would be interesting to further elucidate its function in neuroinflammation and potential as a therapeutic target.

CD200 signaling has been discussed for decades as an important mechanism in neuroinflammatory conditions (Neumann, 2001), but the jury is still out on whether CD200 could be important target to use in ischemic brain injury and neurodegenerative diseases.

Crosstalk between microglia and neuron via fractalkine/CXCR1 has also been studied in experimental stroke models and been a target for neuroprotection. CXCR1 knockout mice subjected to focal brain ischemia was reported by Denes et al. (2008) to surprisingly have reduced IL-1B and TNF production together with reduced infarct size, better recovery and ameliorated neuronal cell death.

Soriano et al. (2002) used the CX3CL1 deficient mice and confirmed the results with the tMCAO model, showing reduced infarct size and reduced mortality when CX3CL1/CX3CR1 signaling was absent.

In stark contrast to these results, when CX3CL1 was administered to naive mice (wt) in combination with pMCAO a reduction in infarct size was reported. In this study, Cipriani et al. (2011) found that the adenosine receptor 1 (A_1_R) was implicate in this neuroprotection and using A1R antagonist or A_1_R^-^/^-^ mice abolished the positive effect of CX3CL1 (Cipriani et al., 2011). However, when they administering CX3CL1 to the CX3CL1 deficient mice they found aggravated the brain ischemia, possibly related to the constitutive lack of CX3CL1 leading to a maladaptation of the CX3CL1/CX3CR1 signaling axis (Cipriani et al., 2011). Clinical data though suggest a potential protective role of CX3CL1, where patients with better clinical outcome after 6 months had higher levels of CX3CL1 in blood plasma (Donohue et al., 2012).

In a recent rat study, Liu et al. (2017) used a pMCAO model to study the effect of CXCR1 inhibitor (day 3–14 systemic administration) and found increased expression of BDNF and NGF, neuroprotection and improved neurology up to 14 days after ischemia.

In view of the dispersed data related to CX3CL1/CX3CR1 and ischemic stroke it is difficult to judge the neuroprotective role of CX3CL1/CX3CR1 signaling and the future possibility to manipulate this pathway pharmaceutically to reduce the sequel of stroke.

## Microglia in Brain Tumors

Prolonged microglia activation has also been observed in the environment of malignant brain tumors. In contrast to what is known in neurodegenerative conditions, tumor-infiltrated microglia exhibit immunologically suppressed phenotypes and they have been demonstrated rather promoting tumor proliferation and progression than exert an anti-tumor activity (Bettinger et al., 2002; Yang et al., 2010; Zhai et al., 2011; Schiffer et al., 2017). The pathological hallmarks of malignant brain tumors involve rapid tumor proliferation, diffuse brain invasion, tumor-induced brain edema and neuronal cell death. Gliomas are the most common and aggressive primary brain tumors that are composed of neoplastic and non-neoplastic cells. The majority of the non-neoplastic cells are tumor-associated macrophages along with fibroblast and endothelial cells (Morantz et al., 1979; Rossi et al., 1987; Simmons et al., 2011). Histopathologic studies of glioblastomas have revealed high number of microglia and macrophages (with peripheral origin) in and around of glioma tissue. This is due to the local release of numerous factors by tumor cells which mediate microglia chemoattraction including chemokines, ligands of complement receptors, ATP and neurotransmitters. Monocyte chemoattractant protein-1 (MCP-1, also known CCL2) was among the first chemoattractant factors, which has been identified to contribute tumor proliferation and progression (Platten et al., 2003; Vakilian et al., 2017). The chemokine stromal-derived factor-1 (SDF-1, also known as CXCL12) has been described as another potent microglia and macrophage recruiting molecules (Wang et al., 2012; Jiang et al., 2013). The growth factor glial cell–derived neurotrophic factor (GDNF), is secreted in mouse and human gliomas, which serves as a strong chemoattractant for microglia (Ku et al., 2013). Colony stimulating factor-1 (CSF-1) is released by glioma cells and acts as a microglial chemoattractant. Blockade of CSF-1R signaling using RNA interference or pharmacological inhibitors have been shown to significantly reduce the number of tumor-associated microglia and glioblastoma invasion (Coniglio et al., 2012). Additionally, CSF-1 is also able to convert microglia into a pro-tumorigenic anti-inflammatory phenotype (Pyonteck et al., 2013). Furthermore, granulocyte- macrophage colony-stimulating factor (GM-CSF) can also serve as a chemoattractant for microglia, as GM-CSF knockdown reduces microglia-dependent invasion in organotypic brain slices and attenuates the gliomas growth *in vivo* (Sielska et al., 2013). Although the tumor-associated microglia are high in numbers, they exert suppressed functions that involves reduced phagocytic activity and defective antigen presentation for cytotoxic and helper T cells activation. Therefore, microglia associated with malignant gliomas appear incapable of inducing an effective anti-tumor T cell response (Wu et al., 2010; Hambardzumyan et al., 2016). Additionally, glioma cells produce numerous anti-inflammatory cytokines (e.g., IL-10, IL-6, TGF-β2, PGE2) that can revert activated microglia to an anti-inflammatory phenotype (Hishii et al., 1995; Pyonteck et al., 2013). For instance, TGF-β2 released by glioma cells inhibits proliferation and secretion of proinflammatory cytokines by microglia and lymphocytes (Suzumura et al., 1993).

In return, microglia secrete tumor proliferation promoting factors including epidermal growth factor (EGF) (Coniglio et al., 2012) and vascular endothelial growth factor (VEGF), TGF-β, arginase-1 (ARG1), and IL-10 (Gabrusiewicz et al., 2011; Li and Graeber, 2012). Additionally, TGF-β released predominantly from microglia are able to enhance tumor growth and invasion as the downregulation the TGF-β type II receptor expression with shRNAs abolished TGF-β-induced glioblastoma invasiveness and migratory responses *in vitro* (Wesolowska et al., 2008). Furthermore, microglia can help increase the spread of tumors by releasing of extracellular matrix degrading enzymes such as MMP-2, MMP-9, and MT1-MMP, into the tumor environment which support tissue remodeling and angiogenesis (Belien et al., 1999; Markovic et al., 2005; Konnecke and Bechmann, 2013).

Neurodegenerative actions of malignant gliomas resemble mechanisms also found in neurodegenerative diseases (e.g., AD, PD, ALS) and brain tumors can affect neuronal survival directly or by microglia-mediated factors. Tumor environments include elevated level of extracellular ATP, which can recruit microglia and macrophages into tumor regions and induce cellular release of inflammatory mediators initiating and sustaining tumor development. ATP is able to activate P2X7 purinergic receptors (P2X7R) expressed on glioma and immune responsive cells (microglia/macrophages). A critical point of ATP signaling in tumors is the prolonged duration in effect due to the inefficient hydrolysis of ATP. The high concentration of extracellular ATP is toxic for neurons and contributes to neuronal cell death (McLarnon, 2017). Additionally, the prolonged activation of microglial P2X7R leads to excessive inflammation by the release of inflammatory factors (IL-1β and TNF-α) and the activation of caspase activity in apoptosis, which can put neuronal survival at risk (Ferrari et al., 1997, 1999). Furthermore, gliomas have been shown to seize neuronal glutamate signaling for their own growth advantage (Savaskan et al., 2015). The cystine/glutamate antiporter xCT is expressed in various malignant tumors including brain tumors (Kim et al., 2001). The protein complex transports Na^+^-independent glutamate out of cells in exchange for cysteine thereby, releases high amounts of glutamate in the extracellular microenvironment. Extracellular glutamate represents a potent signaling molecule and neurotransmitter in the bran tissue and triggers membrane depolarization. However, excessive glutamate release and hence glutamate receptor activation can lead to excitatory neuronal cell death. In brain tumors, the cystine/glutamate antiporter expression is elevated that is consequently causes an increased extracellular glutamate levels in the peritumoral zone. The high extracellular glutamate concentration results in tumor-associated seizures, brain swelling and neuronal damage (Ye and Sontheimer, 1999; Savaskan et al., 2008; Buckingham et al., 2011). In line with this, other experimental and clinical studies have demonstrated that glioma cells secrete high levels of the neurotransmitter glutamate, resulting in neuronal damage and antagonizing ionotropic glutamate receptors alleviate neuronal degeneration in the tumor vicinity and lessen glioma growth *in vivo* (Behrens et al., 2000; Marcus et al., 2010).

## Microglia–Neuron Interaction in Psychiatric Diseases

As previously mentioned, microglia are one of the key mediators of neuroplasticity, acting in the remodeling of synaptic processes and circuitry formation during normal physiological conditions. Therefore, the bidirectional communication between neurons and microglia may be critical for preserving the homeostatic environment in the central nervous system and defects in microglia-neuronal activities have been suggested as potential contributors to neurodevelopmental alterations, resulting in psychiatric disorders such as schizophrenia, bipolar disorder (BD), and depression (Yirmiya et al., 2015; Laskaris et al., 2016).

## Schizophrenia

Schizophrenia is a severe neurodevelopmental disorder characterized by psychosis, apathy and withdrawal, and cognitive impairment, which cause abnormal social behavior and self-care (Mueser and McGurk, 2004; Blank and Prinz, 2013). This disease affects ∼1% of the entire population (Yamamuro et al., 2015), emerges between 16 and 30 years and persists throughout the patient’s life (Mueser and McGurk, 2004). The origin of this disease is still unknown, but some evidences suggest that schizophrenia can arise from an interaction between neurodevelopmental processes, such as prenatal viral infections (Allswede and Cannon, 2018), and environmental factors (Davalieva et al., 2016). Brain structural irregularities have been reported in schizophrenic patients, such as the loss of gray matter in prefrontal, temporal and subcortical structures (Ellison-Wright and Bullmore, 2009; Fornito et al., 2009), the presence of white matter tracts connecting these areas (Bora et al., 2011; Zalesky et al., 2011), as well as an enlargement of ventricle (Olabi et al., 2011). Additionally, this disease presents multiple biochemical irregularities in the dopamine, serotonin, acetylcholine, glutamate, and GABA systems (Mueser and McGurk, 2004), as well as changes in the immune system (Nawa et al., 2000).

In the last few years, microglial cells have appeared as important players in the development of schizophrenia (Brown, 2011). Owing to their participation in inflammatory processes as well as in the modification of neuronal networks (Tremblay et al., 2010; Chew et al., 2013; Zhan et al., 2014b), microglial cells have been proposed as a possible mechanism that participate in the structural brain changes that appear in schizophrenia (Munn, 2000; Monji et al., 2009). Postmortem studies have shown an increased density of microglial cells in the brain of patients with schizophrenia specifically in frontal and temporal, but not in cingulate cortex (Garey, 2010). In addition, positron emission tomography (PET) imaging studies have shown that microglia are activated in these patients (van Berckel et al., 2008; Hercher et al., 2014). These microglia features have been also related with differences in behavior in animal studies, where specifically, schizophrenic rodent models present deficits in pre-pulse inhibition and working memory impairments (Ribeiro et al., 2013).

During the development of the nervous system, the communication between microglia and neuron is fundamental in the synaptic formation and maturation (Penzes et al., 2011) and persistent microglial activation has been used to explain how this process might cause neuronal degeneration and synaptic dysfunction (Monji et al., 2009). CX3CR1 is expressed on microglia during embryogenesis and throughout murine lifespan (Ginhoux et al., 2010; Paolicelli et al., 2011) and it has been proposed as a key mediator of neuron–microglia interactions. In fact, the CX3CR1-knockout mice model presents a reduction of microglia which resulted in deficits of synaptic pruning and a decreased functional brain connectivity (Zhan et al., 2014), as well as behavioral impairments measured by fear conditioning, Morris water maze and motor learning deficits (Rogers et al., 2011). Furthermore, meta-analysis using human blood and brain samples showed a significantly down-regulation of CX3CR1 in schizophrenic patients (Bergon et al., 2015), specifically individuals with Ala55Thr variant of this receptor present more susceptibility to develop this neurological disorder (Ishizuka et al., 2017). Apart from CX3CR1 receptors, secreted Neuregulin 1 (NRG1) can bind to NRG1 receptor on microglia and signal via a type of EGF receptor, ErbB2/3, leading to proliferation and activation of microglia (Calvo et al., 2010). Signaling deficits in NRG1/ErbB3 has been identified in schizophrenia patients (Corfas et al., 2004). Single nucleotide polymorphisms in NRG1; have been associated with psychosis and enlarged lateral ventricles and white matter disruption in schizophrenia (Bousman et al., 2018). Another important mediator between microglia and neuron communication is BDNF, where microglia is one of the central source of BDNF (Ferrini and De Koninck, 2013). Alterations in this gene have been pointed out as a responsible protein for the etiology of schizophrenia (Zhang et al., 2008). Moreover, a single polymorphism (Val66Met) in the BDNF gene has been related to the age of onset of this disorder and the manifestations that persist after durable antipsychotic treatment (Numata et al., 2006).

## Bipolar Disorder

Bipolar disorder is a brain disorder that provokes intense emotion, changes in sleep patterns and activity levels and uncommon behaviors that may go along with mood episodes including symptoms of both manic and depression (Belmaker, 2004). This disorder affects approximately 1% of the population (Newman et al., 2002). The etiology of this psychiatric disorder is still unknown; however, as schizophrenia, the interaction between genes and environment have been related to the pathogenesis of BD (Belmaker, 2004). BD patients showed brain white matter abnormalities (Ganzola and Duchesne, 2017), as well as a decrease in hippocampal (Cao et al., 2016), corpus callosum (Lavagnino et al., 2015), and frontal cortical volumes, specifically in patients with manic (Abé et al., 2015).

Neuroinflammation has been suggested as a possible mechanism involved in mood disorders, such as BD (Perugi et al., 2015; Réus et al., 2015), and alterations in glial markers have been found in postmortem frontal cortex in BD subjects (Rao et al., 2010). Moreover, using PET imaging studies with the microglial tracer ([(11)C]-(R)-PK11195), Haarman et al. (2014) showed an intensification of neuroinflammation in the hippocampus of BD patients. After an insult, microglia can produce proinflammatory cytokines in response to activation via damage-associated molecular patterns (DAMPs) (Heneka et al., 2014). Indeed, numerous meta-analysis in patients with BD showed a significantly increase in peripheral cytokines, such as IL-6 or TNF-α (Goldsmith et al., 2016). Also, serum analysis of BD patients during acute manic/depressive episodes have shown an increased in DAMPs levels (Stertz et al., 2015).

As we have already mentioned, due to their participation in neuroinflammation and remodeling of synapses, microglial cells may contribute in the development of neuropsychiatric disorders, such as BD. Actually, postmortem studies showed a decreased in glial cells in the prefrontal cortex, especially in patients with familial history of mood disorders (Ongur et al., 1998). In particular, it has been suggested that the microglial function is altered in BD patients (Réus et al., 2015). The communication between microglial cells in the brain is mediated by purinergic signaling that includes the P2X purinergic receptor 7 for ATP (P2RX7) (for review, see Fields and Burnstock, 2006). In fact, a single nucleotide polymorphism in the gene that encodes for P2RX7 receptor has been associated with a highest susceptibility for BD pathology (Barden et al., 2006). Likewise, the role of fraktalkines mediated neuron–microglia interaction has been reported in BD (for review, see Pinto et al., 2018). Actually, CX3CR1 expression has been found reduced in monocytes of BD patients (Padmos et al., 2008).

## Depression

Depression is a neuropsychiatric disorder characterized by psychophysiological alterations in affective symptoms (sadness, irritability, low mood, desperation, apathy, anhedonia) that decrease of interest in all daily activities and events (Belmaker and Agam, 2008). According to the World Health Organization, depression affects more than 300 million people worldwide. Conventionally, depressive symptoms have been attributed to an imbalance in the hypothalamic-pituitary-adrenal axis (Sousa et al., 2008) as well as a deregulation in the neuromodulation of neurotransmitters, specially by serotonin (Krishnan and Nestler, 2008). Brain abnormalities have also been found in depressed patients. Thus, postmortem brain studies showed reduced lobar volumes accompanied by a decreased number of synaptic contacts (Lampe et al., 2003; Kang et al., 2012). Moreover, it has been described a loss and a decrease in the size of GABAergic neurons, in occipital, prefrontal and limbic areas (Cotter et al., 2002; Rajkowska et al., 2007; Maciag et al., 2010).

Beyond the classical impairments in the HPA and/or in the neuromodulation by neurotransmitters, depression is also associated with alterations of microglia activity and inflammation (Yirmiya et al., 2015). Rodent models of depression showed significant variations in microglial function and morphology, specifically in brain areas sensitive to chronic stress leading depressive-like behavior, such as hippocampus, prefrontal cortex, amygdala and nucleus accumbens (Tynan et al., 2010; Kreisel et al., 2014). In depressive patients, PET imaging studies revealed a higher level of neuroinflammation in prefrontal cortex, anterior cingulate cortex and insula (Setiawan et al., 2015). Likewise, the communication between microglial cells also may participate in mood disorders. To note, in rodent models, excessive P2X7 receptor activation enhance depressive-like behavior (for review, see Stokes et al., 2015) and in humans, a single nucleotide polymorphism in this gene is associated with the develop of major depressive disorder (Lucae et al., 2006). Besides the alterations in the morphology and function, failures in the microglia–neuron communication may be involved in depression. Some authors showed that the manipulation of this interaction in the CX3CR1 pathway alters the stress reaction and depressive-like behavior (Corona et al., 2010; Milior et al., 2016). Furthermore, the CX3CR1-deficient mouse model showed resistance to stress-induced depressive-like behavior and changes in microglia morphology, suggesting that the hyper-ramification in microglia is controlled by neuron–microglia interaction (Hellwig et al., 2016). Another important mediator between microglia and neurons is BDNF, which has also been linked to depression (Castren, 2014; Kishi et al., 2017). Thus, in a rat model of chronic stress-induced depression, the infusion of this neurotrophin partially reverse the depressive-like behavior (Ye et al., 2011).

## Conclusion

The dynamics of microglia–neuron communication in the healthy brain has attracted great attention in the field of neurobiology/neuroimmunology during the past decades. The bidirectional communication between the two cell-types involves several immunomodulatory factors and signaling pathways including purinergic, neurotransmitter, chemokine, and complement signaling. Many observations indicate that microglia modulates neuronal activity and prune synaptic elements such as dendritic spines in the healthy brain and during neural development. In return, neurons contribute to immune modulation through secreted and membrane associated molecules control microglial phagocytosis, motility and activation. Elucidating the functional significance of the bidirectional microglial–neuronal communication in the healthy brain is important in comprehending how the defects in physiological microglia function could contribute to or even trigger diseases. In brain injuries such as TBI and brain ischemia, microglia–neuron communication is very likely important in both the acute phase, in terms of altered neuroinflammation and cell death, as well as in the chronic phase including the rewiring of neuronal circuits. In neurodegenerative and neuropsychiatric disorders, such as AD, PD, BD depression and schizophrenia, the chronic activation of microglia is likely affecting the pathogenesis and aberrant neuronal signaling. Thus, a deeper understanding of microglia–neuron communication would be important in future therapies for diseases of the brain.

## Author Contributions

ZS, OM, SB, and TD have all designed and drafted sections of the manuscript, as well as significantly contributed to revisions and editing of the final version. OM has also prepared the figures.

## Conflict of Interest Statement

The authors declare that the research was conducted in the absence of any commercial or financial relationships that could be construed as a potential conflict of interest.
